# Adenoviral-Mediated Placental Gene Transfer of IGF-1 Corrects Placental Insufficiency via Enhanced Placental Glucose Transport Mechanisms

**DOI:** 10.1371/journal.pone.0074632

**Published:** 2013-09-03

**Authors:** Helen N. Jones, Timothy Crombleholme, Mounira Habli

**Affiliations:** 1 Center for Fetal Cellular and Molecular Therapy, Cincinnati Children's Hospital Medical Center, Cincinnati, Ohio, United States of America; 2 Colorado Fetal Care Center, Children's Hospital Colorado, Aurora, Colorado, United States of America; John Hopkins University School of Medicine, United States of America

## Abstract

Previous work in our laboratory demonstrated that over-expression of human insulin-like growth factor -1 (hIGF-1) in the placenta corrects fetal weight deficits in mouse, rat, and rabbit models of intrauterine growth restriction without changes in placental weight. The underlying mechanisms of this effect have not been elucidated. To investigate the effect of intra-placental IGF-1 over-expression on placental function we examined glucose transporter expression and localization in both a mouse model of IUGR and a model of human trophoblast, the BeWo Choriocarcinoma cell line.

**Methods:**

At gestational day 18, animals were divided into four groups; sham-operated controls, uterine artery branch ligation (UABL), UABL+Ad-hIGF-1 (10^8^ PFU), UABL+Ad-LacZ (10^8^ PFU). At gestational day 20, pups and placentas were harvested by C-section. For human studies, BeWo choriocarcinoma cells were grown in F12 complete medium +10%FBS. Cells were incubated in serum-free control media ±Ad-IGF-1 or Ad-LacZ for 48 hours. MOIs of 10∶1 and 100∶1 were utilized. The RNA, protein expression and localization of glucose transporters GLUT1, 3, 8, and 9 were analyzed by RT-PCR, Western blot and immunohistochemistry.

**Results:**

In both the mouse placenta and BeWo, GLUT1 regulation was linked to altered protein localization. GLUT3, localized to the mouse fetal endothelial cells, was reduced in placental insufficiency but maintained with Ad-I GF-1 treatment. Interestingly, GLUT8 expression was reduced in the UABL placenta but up-regulated following Ad-IGF-1 in both mouse and human systems. GLUT9 expression in the mouse was increased by Ad-IGF-1 but this was not reflected in the BeWo, where Ad-IGF-1 caused moderate membrane relocalization.

**Conclusion:**

Enhanced GLUT isoform transporter expression and relocalization to the membrane may be an important mechanism in Ad-hIGF-1mediated correction of placental insufficiency.

## Introduction

Fetal growth is reliant upon the proper growth and function of the placenta. In pathological pregnancies altered placental function can lead to inappropriate fetal growth, whether growth restricted or macrosomic, the offspring face increased risk of health problems in both childhood and later life.

Appropriate placental function includes the transport of nutrients from the maternal circulation and both placental and fetal growth is dependent upon transfer of amino acids, fatty acids and glucose. Efficient placental (maternal to fetal) transfer of glucose, the primary substrate for fetal oxidative metabolism, is crucial to sustain the normal development and survival of the fetus in utero because its own glucose production is minimal [1]. Glucose transport is facilitated by members of the SLC2A family, also known as the GLUT transporters, 14 members have been identified within this group to date [2]. Of these, GLUT 1, 3, 8, 9a and 9b expression has been demonstrated in the mammalian placenta [3], [4], [5], [6].

GLUT isoform expression varies depending on the placental cell type studied and may differ depending on the species being studied. In humans GLUT1 is localized to both membranes of the syncytiotrophoblast [3], GLUT3 to syncytiotrophoblast [4] and fetal microvascular endothelial cells [7]. In the mouse, GLUT1 and GLUT3 are localized in the trophoblast and endothelium [8]. Although GLUT8 expression has been confirmed in human and sheep placentas [5] and a large body of evidence indicates that GLUT8 is an intracellular hexose transporter in non-placental cell types [9], localization is currently unpublished in human or mouse placenta. Interestingly, GLUT8 was localized to the plasma membrane following insulin stimulation in mouse blastocysts, a phenomenon not seen in adult cell types. In humans, GLUT9a and 9b appear to be differentially localized to the apical and basolateral membranes of the syncytiotrophoblast respectively [6].

Not only do the GLUT isoforms show different cell specificity but they are also regulated by different effectors and stage of gestation. Hypoxia, insulin, glucose availability, IGF-1, glucocorticoids and CRH [10] have all been shown to regulate GLUT1 in in vivo or in vitro studies. Similarly, hypoxia [11] and CRH [10] also up regulate GLUT3 expression. GLUT1 expression is upregulated towards the end of gestation to meet the demand of the rapidly growing fetus [12]. Brown et al. [13] demonstrated recently that, in humans, GLUT3 expression decreases in the syncytial membranes across gestation with significantly reduced expression towards term.

In pathological pregnancies in humans such as those affected by intrauterine growth restriction (IUGR) or maternal diabetes, alterations in placental glucose transport and isoform expression occur [14], [15], [16]. In genetically- or mechanically-induced animal models of placental insufficiency and intrauterine growth restriction, the function or expression of placental glucose transporters is altered. For example, placental glucose transport was reduced and fetuses were smaller in GLUT3 heterozygous null mice on a calorie restricted diet [17]. Similarly, GLUT8 null mice demonstrated improper embryonic development and implantation leading to an abnormally small growth phenotype into adulthood and have presumably reduced glucose transport [18]. Following maternal sialoadenectomy, EGF deficiency and IUGR occurred as expected and GLUT3 levels were reduced in mouse placenta [19]. When IUGR was induced in sheep by periods of hyperthermia during gestation, placental expression of GLUT8 was reduced [5]. Interestingly, Langdown and Sugden [20] demonstrated enhanced GLUT1 and GLUT3 expression in dexamethasone- induced IUGR in rats which may be linked to the mechanism involved inducing IUGR in that model. To date there are no reports regarding placental expression of GLUT9 in IUGR in humans or animal models.

IUGR can be overcome with treatment with IGF-I, either as protein delivered into the amniotic fluid [21] or as an adenoviral-mediated transgene delivered directly into placenta [22], however the mechanisms are not completely elucidated. We hypothesize that one of the mechanisms how intra-placental delivery of IGF-1 rescues IUGR is via the regulation (or restoration) of GLUT transporters. The current study investigates adenoviral IGF1mediated changes in glucose transport mechanisms in mouse placenta in a surgically induced model of placental insufficiency. To further validate the translational relevance of these findings, we investigated the effects of adenoviral IGF1 mediated changes in glucose transporters in vitro in human trophoblasts using the BeWo Choriocarcinoma model. Glucose transporters 1, 3, 8 and 9 were studied as these isoforms are known to be expressed in the mammalian placenta.

## Materials and Methods

### Ethics Statement

All animal procedures were performed under protocol OCO3024 approved by the Institutional Animal Care and Use Committee of Cincinnati Children's Hospital Medical Center. Under guidelines and approval by the local Institutional Review Board at TriHealth (Bethesda & Good Samaritan Hospitals, IRB Study # 09149-10-002), control, term placentas were obtained at time of C-section from patients signing written, IRB approved consent forms.

### Mouse procedures

Timed pregnant C57 Bl/6J mice were divided into 4 groups:1-Control: Sham operated; 2- uterine artery branch ligation (UABL); 3-UABL with Ad-Lacz 1×10^8^ plaque forming units (pfu); and 4-UABL with Ad-IGF-I 1×10^8^ pfu. At gestational day 18, through laparotomy UABL was performed and treatment was administered as previously described [23). Cesarean-section was performed on day 20, pups and placentas were snap frozen or fixed in PFA for further analysis.

### Human placental tissue

Under guidelines and approval by the local Institutional Review Board (IRB Study # 09149-10-002), normal (control), term placentas were obtained at time of C-section from consented patients and sections of cotyledon were randomly isolated, washed and fixed in 4% PFA for further processing and immunohistochemistry.

### Cell Culture

The BeWo choriocarcinoma cell line was obtained from American Type Culture Collection (ATCC, Manassas, VA, USA). The cells were routinely maintained in 80 cm^2^ flasks at 37°C under 5% CO_2_ and cultured in Ham's F12 nutrient mix supplemented with 10% fetal calf serum and 2% penicillin/streptomycin (Invitrogen, Paisley, Strathclyde, UK). The medium was changed every 2 days and cells were subcultured every 7 days. All cells used were between passages 5 and 15.

### Viral Vectors

Ad-hIGF-1 and Ad-LacZ constructs were obtained from Dr M. Herlyn (Institute for Human Gene Therapy, University of Pennsylvania). Both constructs are replication defective, type 5 adenovirus vectors [23]. Ad-LacZ and Ad-hIGF-1 were given at an MOI of 100∶1 or 10∶1 for 48 hours in serum-free media. Control cells were incubated in serum-free media for the same time frame.

### ELISA

Hu-IGF-1 levels in BeWo cells and mouse placental homogenates were measured in triplicate using a human-specific IGF-1 ELISA (Alpco, Salem, NH) following manufacturer's protocol.

### Immunohistochemistry

Placental tissues were fixed for 24 h at 4°C in 4% paraformaldehyde (or 10% formalin) immediately after dissection, embedded in paraffin, and cut into 5 µm sections. Serial sections were deparaffinized in xylene and rehydrated, sections were incubated in target retrieval solution (Dako Cytomation, Carpathia, CA) at 95°C for 20 minutes, followed by a 20 minute cool down to room temperature. After washing, sections were incubated in 3% hydrogen peroxide to inhibit endogenous peroxidase. Nonspecific binding was blocked by incubation with 5% normal goat serum or 5% normal rabbit serum. Sections were than incubated with anti-GLUT1, 3, and 8 antibodies obtained from Abcam (Cambridge, MA) and anti-GLUT 9a and 9b antibodies kindly provided by Dr. K Moley. After incubation, sections were washed and incubated with appropriate secondary antibodies (Vector Laboratories, Inc., Burlingame, CA). Antibody binding was detected using DAB (Dako Cytomation). Transporter expression was observed by light microscopy (Nikon, Melville, NY).

### Immunocytochemistry

Immunofluorescence staining was performed on BeWo cells on chamber slides following exposure to either control or media containing Ad-hIGF-1 or Ad-LacZ. Cells were fixed in frozen methanol for 5 min. To quench autofluorescence, slides were incubated in 50 mM NH_4_Cl for 10 min at room temperature. To prevent non-specific binding, slides were incubated with 0.2% fish skin gelatin (FSG)/PBS for 5 min at room temperature. Transporter localisation was investigated by using anti-GLUT1, 3, and 8 obtained from Abcam and anti-GLUT 9a and 9b kindly provided by Dr. K Moley. Primary antibody was applied to slides in 0.2% FSG/PBS and allowed to bind overnight at 4°C in a humidified atmosphere. Following rinses in 0.2% FSG/PBS, the anti-rabbit FITC conjugated antibody (Vector Laboratories Inc, Burlingame, CA, USA) was applied to the slides for 1 hour at room temperature in a humidified atmosphere. The slides were rinsed in PBS and deionised water and mounted in Vectashield mounting medium (Vector Laboratories). Cells were observed and images captured using a Nikon microscope and Elements software (Nikon). Controls included the use of anti-rabbit IgG replacing primary antibody during the procedure.

### Reverse Transcription quantitative Polymerase Chain Reaction (RT-qPCR)

Total RNA was isolated from placentas using the RNeasy kit (Qiagen, Valencia, CA) per manufacturer's protocol. cDNA was synthesized from 1 ug of total RNA using the High Capacity cDNA Reverse Transcription kit (Applied Biosystems, Foster City, CA) following suppliers instructions. SYBR green assays were designed to span intron/exon boundaries when possible. Oligonucleotide primers were aligned against the human genome by Primer-BLAST (www.NCBI.org) to ensure specificity. Gene expression was assayed in duplicate, using 1/40^th^ of the cDNA template and 300 nM of forward and reverse primer in a 25 ul Power SYBR Green PCR Master Mix reaction in the Applied Biosystems StepOne-Plus Real-Time PCR System. Gene expression was normalized to TATA-binding protein gene expression. Relative expression values were calculated using the Comparative Ct (ΔΔCt) method [24]. Oligonucleotide primer sequences are as listed in table 1.

**Table 1:** Primer sequences for human and mouse amino acid transporters and housekeeping genes.

Primer Name	5′ to 3′ Primer Sequence	Amplicon Size (bp)	Intron Spanning
Hu SLC2A1 1F	CCT GCA GTT TGG CTA CAA CA	72	Yes
Hu SLC2A1 1R	GTC TGG TTG TAG AAC TCC TCG		
Hu SLC2A3 1F	GTT TCT TTG TTA TTA AAG AAT CAC T	91	Yes
Hu SLC2A3 1R	GTC CAA TTT CAA AAC AGG CCA		
Hu SLC2A8 F	GGG CTG AGC AGA GCT TTC	85	Yes
Hu SLC2A8 R	AAG GCC ATC AGG GAG ACG		
Hu SLC2A9 F	CAG CGA AAA AGA AAT TGG ACT G	84	Yes
Hu SLC2A9 R	TAG CCG TAG AGG AAG GAG G		
Hu TBP F	GAA CCA CGG CAC TGA TTT TC	77	Yes
Hu TBP R	TGC CAG TCT GGA CTG TTC TTC		
Mo SLC2A1 1F	TGT GCT CAT GAC CAT CGC	90	Yes
Mo SLC2A1 1R	AAG GCC ACA AAG CCA AAG AT		
Mo SLC2A3 1F	TCT CTC TTT CTG GCC TGG AG		Yes
Mo SLC2A3 1R	TCT CTG CTG GAT GAC GTG AG		
Mo SLC2A8 F	GCT GAA AAC TTT GGA AGA CAG A		No
Mo SLC2A8 R	CTT CAG ATG CCA CAG ACT CC		
Mo SLC2A9a F	ACC TGT CCA GGT GAG AAA GA		Yes
Mo SLC2A9a R	GTG GTC TTC CAG CGT CAC		
Mo SLC2A9a F	TGG TGC TCA CCA TCA TCT C		Yes
Mo SLC2A9a R	ATC ACC TTC CAT CGG ATC TC		
Mo RPS20 F	ATG AGA GGC CAT CAT TTG CC		Yes
Mo RPS20 R	GCG TGG TAT TCA CGT AGG TC		

### Western blotting

BeWo cells were washed three times in ice-cold BSS at pH 7.4. Cells were homogenised and sonicated. Total protein (30 µg) was separated on a 4–20% Tris–glycine gel and transferred to Hybond-ECL nitrocellulose membrane using the Iblot system (Invitrogen, Carlsbad). Membranes were incubated with the appropriate primary antibody overnight at 4°C and an HRP-anti-rabbit IgG antibody (Sigma-Aldrich, St. Louis, MO) for 2 hours at room temperature. Protein bands were visualised using Super Signal West Pico Chemiluminescent substrate (Pierce, Rockford, IL, USA). Protein expression was quantified using densitometry normalised against β-actin for loading controls and internal controls for blot comparison.

### Data presentation and statistics

Data are presented as means ±SEM. Statistical analysis was performed using ANOVA with post hoc Tukey's test *p<0.05, **p<0.01, ***p<0.005.

## Results

### IGF-1 Expression

To ensure transgene expression and increase in Human IGF-1 levels, BeWo media and mouse placental homogenates were analyzed by ELISA. BeWo cells exposed to Ad-hIGF-1 (MOI 100) showed a doubling in IGF-1 secretion when normalized to total protein compared to control and Ad-LacZ (MOI 100) treated cells (Hu-IGF-1 74.7±7.0 ng/ml vs Ad LacZ 35.4±1.8 vs Ctrl 38.8±4.2, p<0.001, n>4 passages). Human IGF-1 was not detected in control mouse placental samples, however in Ad-huIGF-1 treated placentas 27.4 ng/mg tissue were detected (n = 5 placentas).

### Mouse placental GLUT mRNA expression

**Figure 1:**
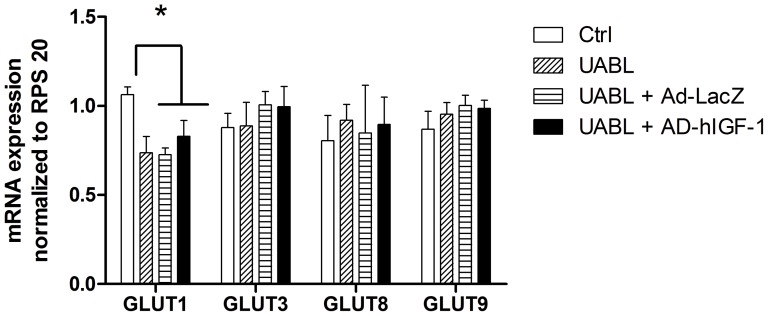
Summary of GLUT1, 3, 8 and 9 mRNA expression in control, UABL, Ad-hIGF-1 and Ad-LacZ treated mouse placentas. ANOVA, Post hoc Tukeys test * p<0.05, n>3 for each treatment

All of the GLUT isoform mRNAs investigated in this study (GLUT1, 3, 8, 9a, 9b) were expressed in the mouse placenta as confirmed by real-time PCR (figure 1, n>3 biological replicates for each treatment). Mouse placental GLUT1 mRNA expression was reduced by 30% (p<0.01, n>5) in the UABL group compared to sham but remained at this reduced level following Ad-hIGF-1 treatment. However, GLUT3, 8, 9a or 9b mRNA expressions were not changed by either uterine artery branch ligation or exposure to Ad-hIGF-1.

### Mouse placental GLUT protein expression

**Figure 2:**
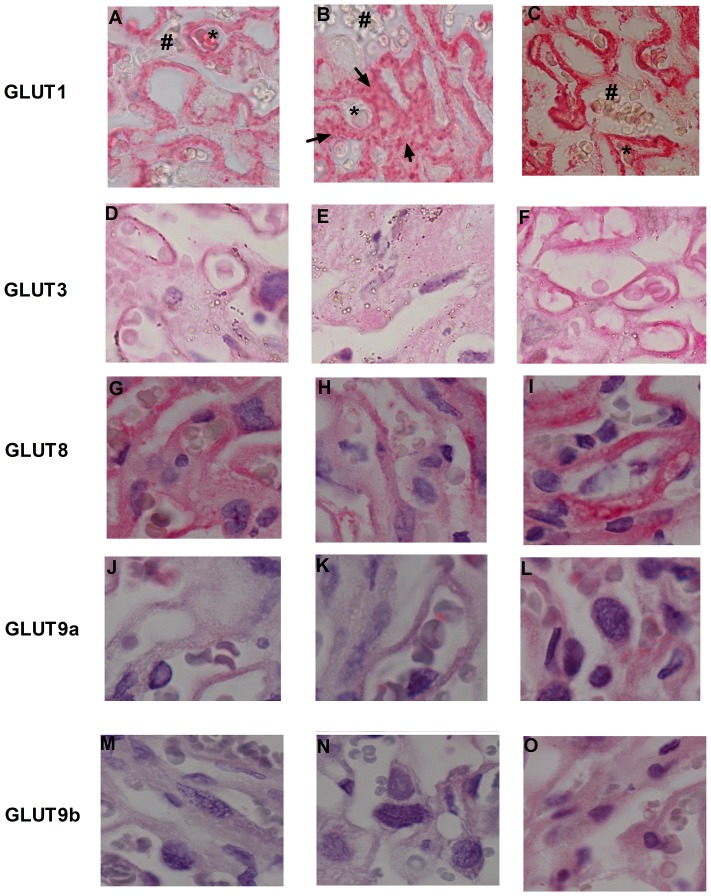
Representative micrographs of GLUT1 (A,B,C), GLUT3 (D,E,F), GLUT8 (G,H,I), GLUT9a (J,K,L) and GLUT9b (M,N,O) in mouse placental labyrinth in the 3 treatment groups, Sham, UABL and Ad-hIGF-1 respectively. 100× magnification. ST identifies syncytiotrophoblast, * identifies a fetal villous vessel, # identifies a maternal blood sinus. Micrographs are representative of 3 biological replicates for each treatment group.

GLUT1, 8 and 9a proteins were localized to the syncytiotrophoblast in the sham mouse placenta. GLUT1 and 8 protein expression was reduced by UABL (fig.2B, H). GLUT9a/9b expression did not change with UABL. Interestingly only GLUT8 and GLUT9a/b, but not GLUT1, showed increased protein expression with Ad-hIGF-1 treatment (fig.2I, L, O) compared to those from the UABL group (fig.2H, K, N). Interestingly, GLUT9b protein expression was not seen in the labyrinth zone, but was present in the Junctional and Decidual zones in the sham and UABL samples (fig 2 M,N).However following Ad-IGF-1 treatment GLUT9b expression was seen in the syncytium (fig.2 O). GLUT3 expression appears to be mostly localized to the fetal endothelial cells in both control (2D) and IGF-1 treated placentas (2F), however there is an apparent lack of GLUT3 expression in the placentas following uterine artery branch ligation (fig 2E).

### Human placental GLUT8 localization

**Figure 3:**
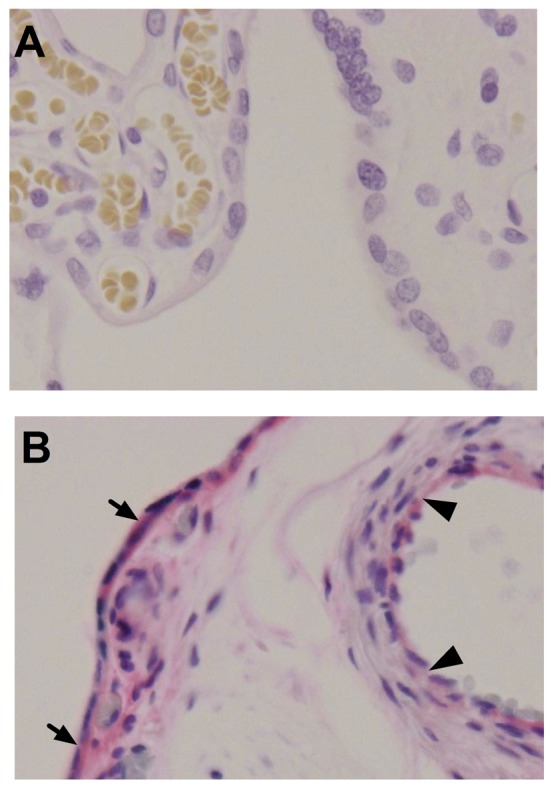
Localization of GLUT8 in human placenta. A) Rabbit IgG control demonstrating no staining in human placenta. B) GLUT8 is expressed in the syncytiotrophoblast (closed arrows) and the villous vessel endothelium (black arrowheads) in normal, term, human placenta.

Prior to investigating the effects of Ad-hIGF-1 in a model of human trophoblast in vitro, we confirmed the location of GLUT8 expression in the human placenta. Figure 3 demonstrates the expression of GLUT8 in normal, term placenta in the syncytiotrophoblast and also some evidence of expression in the fetal endothelial cells.

### In Vitro studies of Ad-hIGF-1 in BeWo cells

**Figure 4:**
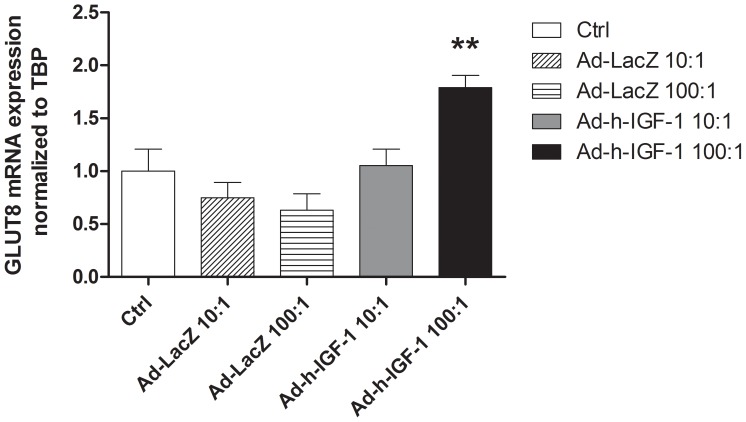
GLUT8 mRNA expression is significantly increased following exposure of BeWo cells to Ad-IGF-1 at an MOI of 100∶1 for 48 hours. ANOVA, Tukeys posthoc test ***p<0.005, n = 6 replicates

The glucose transporters GLUT1, 3, 9a or 9b showed no changes in mRNA expression with Ad-hIGF-1 or Ad-LacZ transfection at MOI of 10∶1 or 100∶1 (data not shown). In contrast, GLUT8 mRNA expression was significantly increased following treatment with Ad-hIGF-1 (MOI 100∶1) compared to untreated and viral-control treated cells (figure 4).

**Figure 5:**
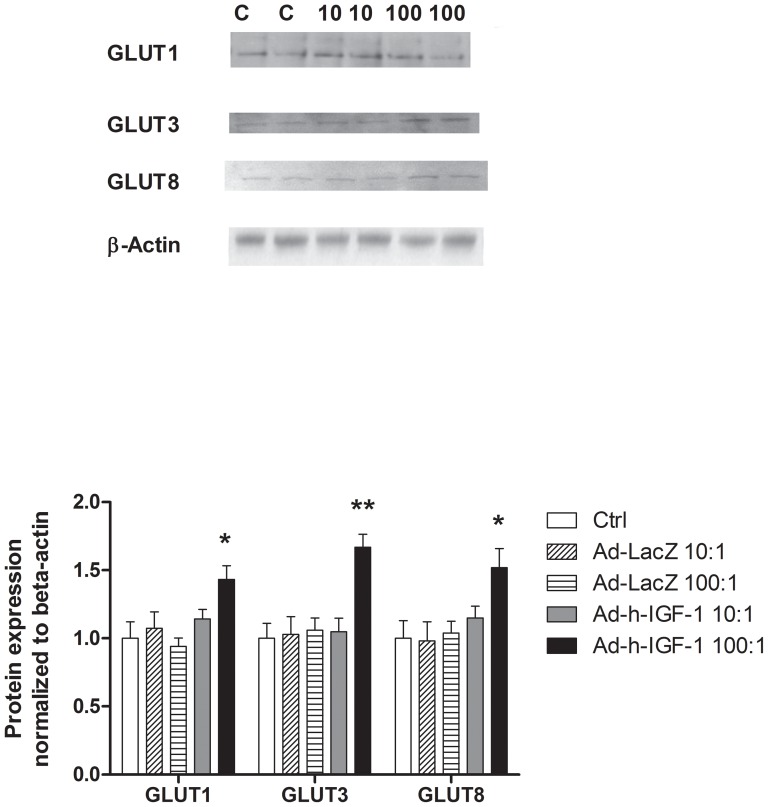
GLUT expression levels in BeWo cells. A:Representative Western blots of GLUT1, 3, and 8 in BeWo cells treated with control media (C), Ad-hIGF-1 MOI 10∶1 (10), Ad-hIGF-1 MOI 100∶1 (100) for 48 hours. B: Summary of GLUT1, 3, 8 protein expression. ANOVA, Tukeys posthoc test, *p<0.05, ** p<0.01, n>4 replicates for each treatment.

**Figure 6:**
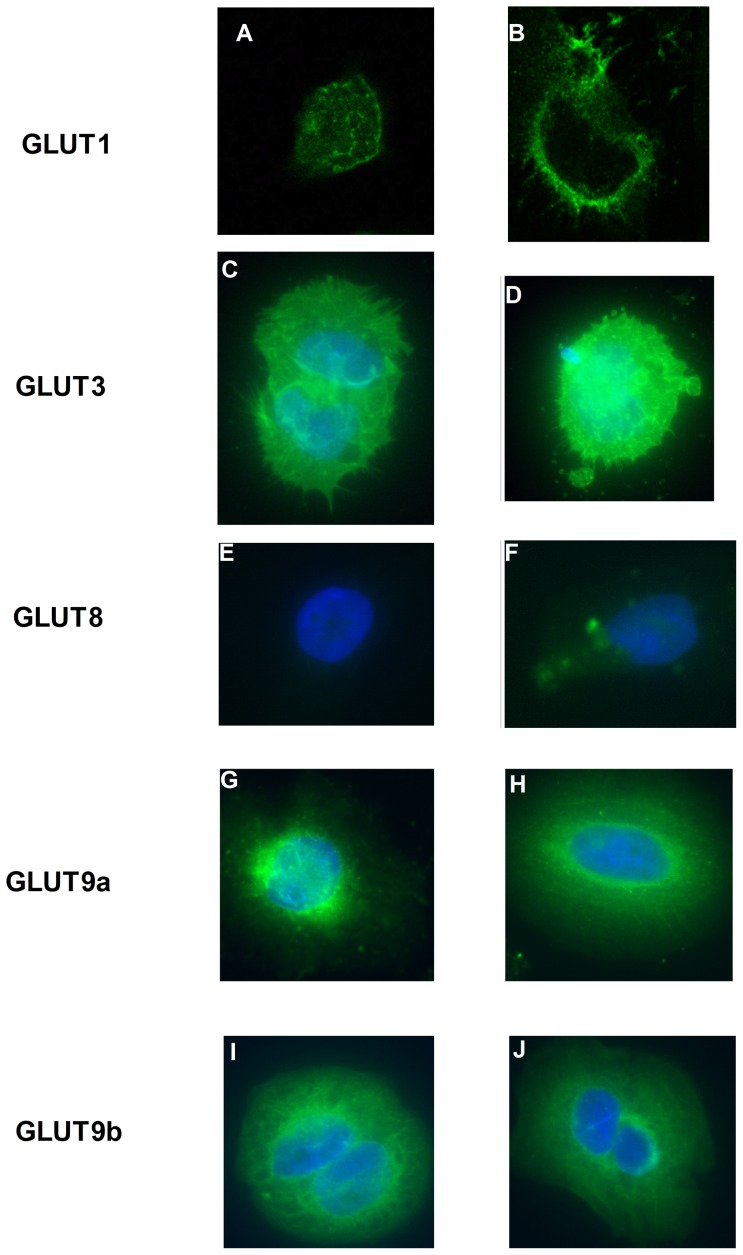
GLUT1 localization is shown in control cells (A) and Ad-hIGF-1 (B) treated BeWo cells1. GLUT3 is localized to both the perinuclear region and the cell membrane in both control (C) and Ad-hIGF-1 (D) treated BeWo.GLUT8 protein expression is minimal in control BeWo (E) but increased following exposure to Ad-hIGF-1 (F). Under control conditions GLUT9a remains perinuclear (G) but following exposure to Ad-hIGF-1 -localizes to the cell membrane (H). GLUT9b is distributed throughout the cell membrane in control (I) and Ad-hIGF-1 (J) treated BeWo cells. Images are representative of at least 3 different passages of BeWo cells.

GLUT1 protein expression was significantly increased following exposure to Ad-hIGF-1 (MOI 100∶1) compared to untreated and viral-control treated cells (figure 5). Immunocytochemistry for GLUT1 also demonstrated localization of the transporter to the cell membrane following 48 hours incubation with Ad-hIGF-1 (MOI 100∶1, figure 6) compared to control cells. GLUT3 protein expression was increased following exposure of the BeWo Choriocarcinoma cells to Ad-hIGF-1 (MOI 100∶1, figure 5) but there was no apparent change in protein localization (figure 6). Similarly, GLUT8 protein expression was increased by exposure of the BeWo cells to Ad-hIGF-1 at MOI 100∶1 compared to untreated or viral-control cells (figure 5 & 6). GLUT9a and 9b were highly expressed in BeWo cells. Under control conditions 9a remains perinuclear (figure 6) but following exposure to Ad-hIGF-1, it relocalizes to some extent to the cell membrane. In contrast GLUT9b is distributed throughout the cell membrane in control and Ad-hIGF-1 treated cells (figure 6).

## Discussion

In the current study we investigate the alteration of placental glucose transport mechanisms following Ad-hIGF-1 treatment in an in vitro model of human trophoblast and a mouse model of placental insufficiency. Maintaining fetal carbohydrate supply is vital for appropriate fetal growth and has been shown to be diminished in placentas associated with IUGR [3]. Therefore, the restoration of appropriate placental glucose transport and avoidance of fetal hypoglycemia should be an essential requirement of future placental gene therapy treatments for Intra-Uterine Growth Restriction. In the current study we investigate the alteration of placental glucose transport mechanisms following Ad-hIGF-1 treatment in an in an in vitro model of human trophoblast and a mouse model of placental insufficiency. Furthermore, we demonstrate that IGF-1 treatment involves the regulation of multiple different glucose transporters in the placenta.

This is the first study to investigate the effects of Ad-hIGF-1 on glucose transport in the placenta either in vivo or in vitro. The treatment of both the BeWo cell line and mouse placenta with Ad-hIGF-1 leads to over-expression of IGF-1 protein. Importantly, there were differences in the regulation of the expression of glucose transporter isoforms following Ad-hIGF-1 treatment between the in vivo and in vitro studies. Using the viral control Ad-LacZ, we have demonstrated that the adenovirus itself does not influence glucose transporter expression, suggesting that the effects are hIGF-1 transgene mediated.

In our mouse model of placental insufficiency (PI), GLUT1 RNA and protein expression was reduced in contrast to published studies in IUGR in humans [3] but in agreement with other animal models of PI or fetal growth restriction. Interestingly, there is staining seen in the fetal mesenchyme in the UABL group which is not present in the sham or Ad-IGF-1 treated animals, suggesting perhaps a different response when other cell types are ‘stressed’ in placental insufficiency. Over-expression of IGF-1 seems to affect GLUT1 expression differently in the two models. IGF-1 over-expression does not appear to increase GLUT1 expression in the mouse model in the syncytiotrophoblast. However, over-expression of IGF-1 seems to affect GLUT1 expression differently in the human trophoblast cell line, where it results in increased GLUT1 expression. The impact of IGF-1 over-expression on GLUT3 also differed between the two models. These may be species differences or may be due to the differences in gestational time. In human, GLUT3 is thought to be more important in early gestation, as reflected by the BeWo cell line, and not in term trophoblast (as represented by the mouse model). Furthermore, the regulation of GLUT3 in the fetal endothelial cells in vivo may be associated with the regulation of glucose transfer from the placenta to the fetus and when supplies are reduced, as in the UABL model, the placenta may reduce transfer to the fetus and retain glucose for placental metabolism.

Our results demonstrate for the first time the expression and localization of GLUT8 in the mouse and confirm it in the human placenta and the BeWo Choriocarcinoma cell line. Interestingly, in the human placenta, expression was seen in both the syncytiotrophoblasts and the fetal endothelial cells, suggesting an involvement of GLUT8 in trans-placental transfer of glucose. The effect of placental insufficiency on GLUT8 expression or function in humans is currently unknown and requires further investigation. In our PI mouse model the expression of GLUT8 was reduced concurring with studies in a sheep model of fetal growth restriction [5], together these studies indicate a strong need to evaluate placental GLUT8 involvement in human IUGR. In both the mouse placenta and human BeWo cell line, over-expression of IGF-1 mediates an increase in GLUT8 protein expression with some indication of membrane localization in the BeWo cell line but not the mouse placenta. This membrane relocalization is in agreement with the insulin-stimulated membrane recruitment previously shown in mouse blastocysts. However, in other cell types GLUT8 is thought to be involved in intracellular hexose transport with no evidence of membrane localization [9]. Our study further indicates a difference in the role of GLUT8 in glucose transport may exist between fetal and adult life.

In contrast to the study by Bibee et al. in the human placenta, the two isoforms of GLUT9, a and b, do not appear to preferentially localize to opposite membranes of the placental syncytium in the mouse. Interestingly the GLUT9b isoform is very lowly expressed in the murine labyrinth under normal conditions but shows some expression in the Junctional zone. However expression occurs in the labyrinth following IGF-1 over-expression. The difference in arrangement of the mouse syncytial layers compared to the single syncytium in humans may also account for a lack of targeting of these isoforms in the murine placenta. Furthermore, fetal growth restriction is not associated with altered expression of these two isoforms of GLUT9 in this model of placental insufficiency. Interestingly, our study demonstrates the apparent increase in both of the GLUT9 isoforms following IGF-1 over-expression in the mouse placenta and the BeWo cell line, however, only GLUT9b in the BeWo cells demonstrated relocalization to the membrane. This is the first report of IGF-1 regulation of the GLUT9 isoforms and, in the mouse, may indicate the recruitment of alternative glucose transporters by IGF-1 treatment following placental insufficiency when GLUT1 remains at reduced levels.

In this study we see a lack of correlation between RNA and protein expression levels following Ad-IGF-1 treatment in both models. One explanation for this could be based on the initiation of translation by IGF-1, known to be an important regulatory mechanism in multiple cell types when treated with IGF-1. Further studies into the signaling mechanisms regulating the GLUT8 and GLUT9 transporters will aid characterization of these isoforms. The use of the BeWo Choriocarcinoma, while still a convenient and well-established model of trophoblast may be a limitation of this study and future studies utilizing primary human trophoblast may also aid in identification of IGF-1 regulatory mechanisms in the placenta.

Despite differences seen between the two models used in this paper we conclude that the restoration of birthweight in mice treated by intra-placental Ad-hIGF-1 delivery may be in part due to alterations in placental glucose transporter mechanisms. Maintaining an adequate glucose supply, while vital to fetal growth, may not be the only mechanism responsible for the restoration of fetal growth in this model and the authors are also investigating alterations in other placental nutrient transport systems, such as amino acids and alterations in placental structure beyond the scope of this paper. Furthermore, we have demonstrated IGF-1 regulation of GLUT8 and GLUT9a and b both in vitro and in vivo. Identification of the involvement of these different isoforms in human IUGR will be necessary prior to development of human placental gene therapy.
